# Diagnosis potential of subarachnoid hemorrhage using miRNA signatures isolated from plasma-derived extracellular vesicles

**DOI:** 10.3389/fphar.2023.1090389

**Published:** 2023-02-13

**Authors:** Bin Sheng, Niansheng Lai, Tao Tao, Xiangxin Chen, Sen Gao, Qi Zhu, Wei Li, Qingrong Zhang, Chunhua Hang

**Affiliations:** ^1^ Department of Neurosurgery, Nanjing Drum Tower Hospital, The Affiliated Hospital of Nanjing University Medical School, Nanjing, China; ^2^ The Translational Research Institute for Neurological Disorders of Wannan Medical College, Department of Neurosurgery, The First Affiliated Hospital of Wannan Medical College (Yijishan Hospital of Wannan Medical College), Wuhu, China; ^3^ Department of Neurosurgery, Nanjing Drum Tower Hospital, Clinical College of Nanjing Medical University, Nanjing, China

**Keywords:** subarachnoid hemorrhage (SAH), extracellular vesicles (EVs), miRNA, biomarker, prognosis

## Abstract

The diagnosis and clinical management of aneurysmal subarachnoid hemorrhage (aSAH) is currently limited by the lack of accessible molecular biomarkers that reflect the pathophysiology of disease. We used microRNAs (miRNAs) as diagnostics to characterize plasma extracellular vesicles in aSAH. It is unclear whether they can diagnose and manage aSAH. Next-generation sequencing (NGS) was used to detect the miRNA profile of plasma extracellular vesicles (exosomes) in three patients with SAH and three healthy controls (HCs). We identified four differentially expressed miRNAs and validated the results using quantitative real-time polymerase chain reaction (RT-qPCR) with 113 aSAH patients, 40 HCs, 20 SAH model mice, and 20 sham mice. Exosomal miRNA NGS revealed that six circulating exosomal miRNAs were differentially expressed in patients with aSAH versus HCs and that the levels of four miRNAs (miR-369-3p, miR-410-3p, miR-193b-3p, and miR-486-3p) were differentially significant. After multivariate logistic regression analysis, only miR-369-3p, miR-486-3p, and miR-193b-3p enabled prediction of neurological outcomes. In a mouse model of SAH, greater expression of miR-193b-3p and miR-486-3p remained statistically significant relative to controls, whereas expression levels of miR-369-3p and miR-410-3p were lower. miRNA gene target prediction showed six genes associated with all four of these differentially expressed miRNAs. The circulating exosomes miR-369-3p, miR-410-3p, miR-193b-3p, and miR-486-3p may influence intercellular communication and have potential clinical utility as prognostic biomarkers for aSAH patients.

## Introduction

Aneurysmal subarachnoid hemorrhage (aSAH), which generally results from ruptured aneurysms, is a clinical syndrome with nearly 45% mortality and morbidity in approximately 0.1‰ of individuals worldwide every year, and 5% of cases involve subarachnoid hemorrhage in the cerebrovasculature. aSAH occurs in patients aged 50–55 years at more than 5% of the total incidence of stroke (Nasra et al., 2022; Zeineddine et al., 2022). Initial hemorrhaging that causes early brain injury (EBI) and early cerebral vasospasms is a vital prognostic determinant and is possibly associated with delayed cerebral ischemia (DCI) (Sheng et al., 2018a; Takeuchi et al., 2021; Zhang X. et al., 2021). Recently, researchers have found that EBI after SAH may be a leading factor contributing to unfavourable outcome in SAH (Ahn et al., 2022; Tao Q. et al., 2022). Therefore, it is essential to understand the pathophysiological changes in the early phase after aSAH, including microvascular filling defects, inflammation, and microarterial narrowing (Helbok et al., 2015; Huang et al., 2017). Therefore, a reliable, economical, and non-invasive approach is needed to provide screening of patients and contribute to improving the prognoses of patients with aSAH.

Extracellular vesicles, including exosomes and microvesicles, are important mediators of intercellular communication and used as biomarkers for disease (Badimon et al., 2022; Clancy and D'Souza-Schorey, 2022). In this study, we detected extracellular lipid vesicles of 30–150 nm in diameter, most of which consisted of exosomes, in plasma from patients with subarachnoid hemorrhage, and the subsequent analyses were primarily in exosomes. Exosomes can cross the blood–brain barrier, where they specialize in long-distance intercellular communication and facilitate the transfer of proteins, lipids, functional mRNAs, and miRNAs for the subsequent expression of proteins at their target cells (Goetzl, 2020; Zhang L. et al., 2021). A similar study reported that exosomes from peripheral blood could be enriched and used for detecting proteins, lipids, and nucleic acids (Kanninen et al., 2016). These circulating exosomal miRNAs could be ideal biomarkers to reflect the pathological progression of aSAH.

MicroRNAs (miRNAs) are a family of non-coding RNAs of 17–24 nucleotides that regulate the expression of multiple target genes at the post-transcriptional level (Baluni et al., 2022). Previous studies of miRNAs as biomarkers of aSAH have been reported (Bache et al., 2020; Kalani et al., 2020). Other studies have demonstrated significant differential expression of miRNAs in human cerebrospinal fluid after aSAH (Bache et al., 2017; Stylli et al., 2017). Nevertheless, the variety and characteristics of miRNAs in the plasma of patients with aSAH remain unknown.

In this study, we determined the expression profiles of plasma exosomal miRNAs in post-aSAH patients and healthy controls using next-generation sequencing (NGS). We then determined the feasibility of measuring differential expression levels of miRNAs in plasma from aSAH patients and in a SAH mouse model. Finally, we estimated the relevance of differential miRNA expression to prognosis. The purpose of the study was to determine the clinical significance and prognostic value of plasma exosomal miRNAs in aSAH.

## Materials and methods

### Human subjects and animal studies

Study participants were enlisted from the Department of Neurosurgery, The First Affiliated Hospital of Wannan Medical College, China. The study was compliant with the Declaration of Helsinki. All participants or valid proxies signed an informed consent form prior to inclusion. All experiments were approved by the hospital ethics committee (No. 2019–86) and were performed in accordance with the National Institutes of Health Guidelines for the Care and Use of Animals.

Healthy adult male C57BL/6 J mice (8–10 weeks) weighing 22–25 g were used in all experiments. The mice were purchased from the Animal Center of Zhejiang Province, China. All the mice were kept in temperature- and humidity-controlled animal quarters with a 12-h light/dark cycle. Every effort was made to minimize the number of animals used, as well as their suffering.

### Study design

**FIGURE 1:**
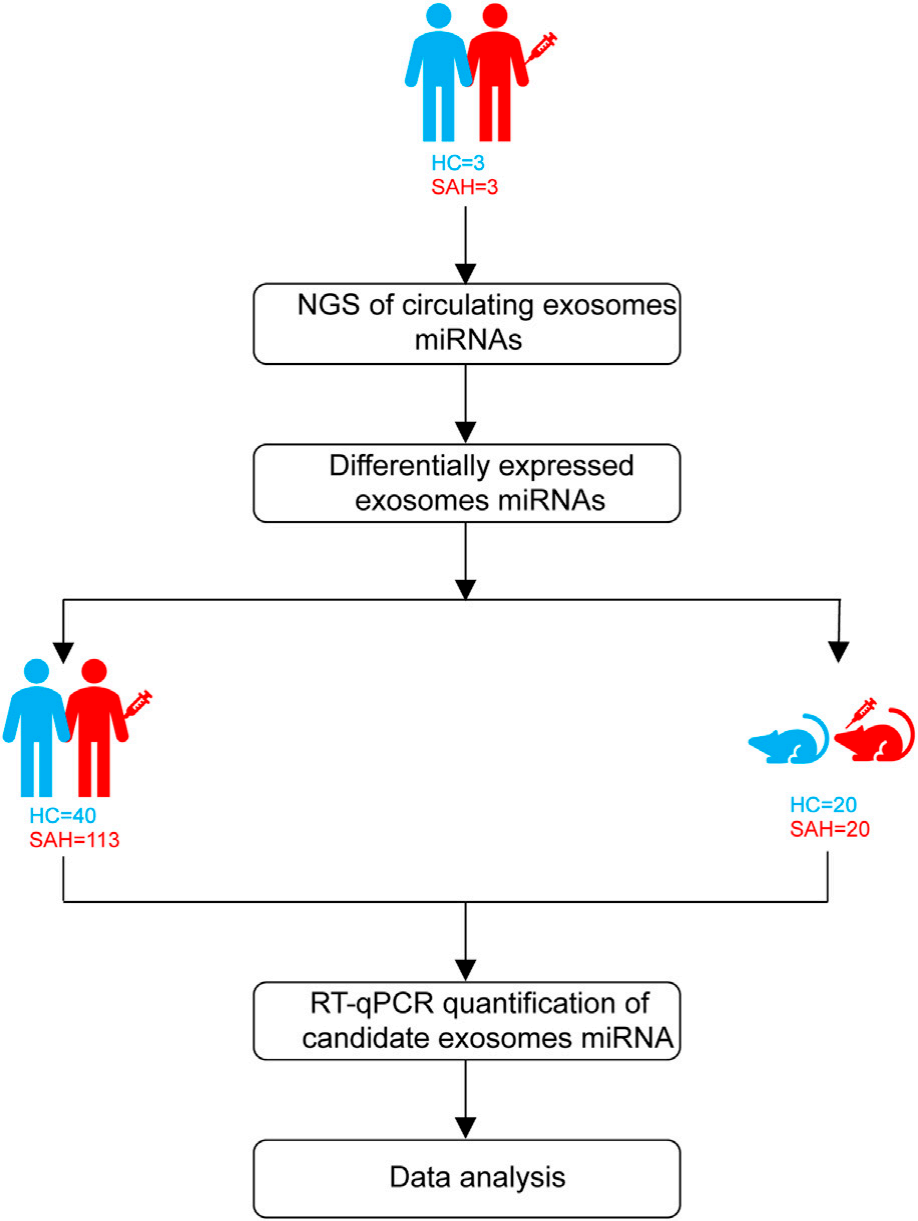
Schematic diagram of the study design.

Patients with aSAH were admitted from April 2020 to February 2021. The study design is shown in Figure 1. First, exosomal miRNA profiles were generated for three sets of plasma (three aSAH patients and three healthy controls) by NGS, and the results were confirmed by RT-qPCR. To analyze the miRNA NGS results, 20 serum samples (10 aSAH and 10 healthy controls) were selected by RT-qPCR. Subsequently, the candidate miRNAs were further verified by RT-qPCR in plasma samples from 113 aSAH patients and 40 healthy controls. Finally, in the SAH mouse model study, the validated miRNAs were tested in the plasma of SAH-model mice.

Neurological outcomes were evaluated by the modified Rankin Scale (mRS) at one year post-aSAH using outpatient outcomes. At the end of the follow-up period, patients with mRS scores of 0–2 were considered to have good outcomes, and those with mRS scores of 3–6 were considered to have poor outcomes (Limin et al., 2022).

### Sample processing

The study involved 193 plasma samples from 113 aSAH patients, 40 healthy controls, 20 SAH mice, and 20 sham control mice. Blood was collected within 24 h of admission, and each sample was obtained in the fasted state. Plasma fractionation was performed within 2 h of obtaining whole blood. The whole blood was centrifuged at 500 rpm and 4°C for 10 min. The upper layers were transferred into RNase/DNase-free 1.5-mL EP tubes and centrifuged further at 3000 rpm at 4°C for 10 min. Plasma aliquots were stored at −80°C for further analysis.

### SAH model

Following our previous studies (Tao T. et al., 2022), briefly, animals were anesthetized with 3% inhaled isoflurane and maintained with 1.5% isoflurane during surgery. Meloxicam (5 mg/kg) was administered subcutaneously after anesthesia for pain relief. Mice were placed on a heating pad to maintain their body temperature throughout the procedure, as monitored by an anal temperature sensor. The external carotid artery was ligated and divided slightly at the bifurcation, with an indwelled knot. The vessel was cut close to the fractured end, and a prelabeled monofilament was inserted from the ECA backward, through the internal carotid artery to the middle cerebral artery bifurcation. In the subarachnoid hemorrhage group, the monofilament was quickly inserted into the pointed nylon monofilament (6–0), with a slight breakthrough feeling, and then pulled out; in the sham group, the monofilament was removed directly. The knots were tied to prevent bleeding. One milliliter of saline was injected intraperitoneally. After surgery, the mice were put into the animal postoperative nursing room until they fully awakened; they were fed jelly to provide energy and water. Finally, blood was sampled and subjected to further analysis after SAH mice were sacrificed at the indicated time points.

### Isolation of exosomes from plasma samples

Exosomes were isolated from plasma using the exoEasy Maxi Kit (Qiagen, Valencia, CA), following the manufacturer’s instructions. Briefly, syringe filters (EMD Millipore, Burlington, MA) were used to filter plasma samples to exclude particles larger than 0.8 μm. One milliliter of Buffer XBP was added to 1 mL of plasma samples, mixed for 5 min, centrifuged at 500 *g* for 1 min, and then bound to exoEasy membrane spin columns. The bound EVs were washed with Buffer XWP by centrifugation at 5,000 *g* for 5 min and eluted with 400 μL Buffer XE by centrifugation at 5,000 *g* for 5 min to collect the eluates, after which they were ready for further analysis. All processes were performed at room temperature.

### Exosome identification

For exosome identification, transmission electron microscopy (TEM, Hitachi HT7700, Tokyo, Japan) was performed to observe the morphologies of precipitated particles. Briefly, we extracted 5 μL of the previous eluates and diluted them to 10 μL. Then, we extracted 10 μL samples onto copper disks for 1 min and used filter paper to remove the floating material. Subsequently, 10 μL phosphotungstic acid was dropped on copper disks for 1 min, and the floating material was removed with a filter paper. After drying for several minutes at room temperature, we examined the samples using TEM. The size and concentration of the particles were measured and analyzed using the NanoFCM instrument (NanoFCM Inc. China). The levels of CD63 and Alix were measured by Western blot, and the exosome biomarkers CD9 and CD81 were measured using the NanoFCM instrument.

### Isolation and concentration measurement of exosome proteins

The bicinchoninic acid (BCA) protein assay kit (Beyotime, P0010) was used to measure the concentration of exosomes. Briefly, the isolated exosomes were melted at 37°C. We quickly added equal volumes of RIPA lysis buffer, and then mixed and split on ice for 30 min. We prepared a BCA protein concentration standard sample and then added it to the BCA mixture and mixed. After incubating for 30 min at 37°C, optical density was measured at 570 nm on a microplate reader. The protein concentration of the sample was calculated according to the standard curve.

### Western blot analysis

Ten percent sodium dodecyl sulfate–polyacrylamide gels were used to separate molecular weight markers (5 µ/lane, Thermo Scientific, USA) and protein samples (20 µg/lane), which were then electrophoretically transferred onto polyvinylidene difluoride membranes (Millipore Corporation, USA). Then, 5% non-fat milk and primary antibodies were used to block membranes for 1 h at room temperature, and blots were incubated in 5% BSA overnight at 4°C. Mouse anti-CD63 and rabbit anti-Alix (all 1:1,000, Abcam) were used as primary antibodies. Corresponding HRP-conjugated anti-rabbit or anti-mouse (1:10,000, Pierce) secondary antibodies were incubated for another 2 h under the same conditions. Bands were visualized with an enhanced chemiluminescence kit.

### Flow NanoAnalyzer studies

We diluted 20 µL exosome to 60 μL and then added 20 µL fluorescent marker antibodies (CD9 and CD81) to 30 µL dilution. We incubated for 30 min at 37°C after mixing. We added 1 mL pre-cooled PBS and then ultracentrifuged for 70 min at 110,000 x *g* and 4°C. After removing supernatants, we repeated centrifugation. We again removed supernatants, resuspended in 50 µL pre-cooled 1 x PBS, and analyzed using the NanoFCM NanoAnalyzer (NanoFCM, China) as per manufacturer instructions.

### miRNA NGS analysis

miRNA profiling of exosomes was performed using NGS. A detailed NGS analysis is described in the Supplementary Material.

### RNA isolation and RT-qPCR of miRNAs

Total RNA was extracted from exosomes using the exoRNeasy Serum/Plasma Midi Kit for exosomes (Qiagen, Valencia, CA) and QIAzol for tissues (Qiagen, Valencia, CA), following the manufacturer instructions. cDNA was synthesized using the miRcute Plus miRNA First-Strand cDNA Synthesis Kit (Tiangen Biotech). The quantification of miRNA was performed using the miRcute Plus miRNA qPCR Detection Kit (SYBR Green) according to the manufacturer’s protocol. A detailed experimental protocol is described in the Supplementary Material.

### Statistical analysis

Data were analyzed using MedCalc version 15.0 (MedCalc, Belgium). Data were presented using the Mann–Whitney U test. Spearman’s rank correlation coefficient analysis was used to calculate correlations among the variables. Receiver operating characteristic (ROC) curves were constructed to determine the optimal thresholds of miRNAs to predict SAH outcomes. A multivariate logistic regression model was analyzed to determine factors independently predicting mRS, after adjusting for risk factors that reached *p* < 0.1 in the univariate analysis. *p* < 0.05 was considered significant.

## Results

### Exosome characterization

**FIGURE 2:**
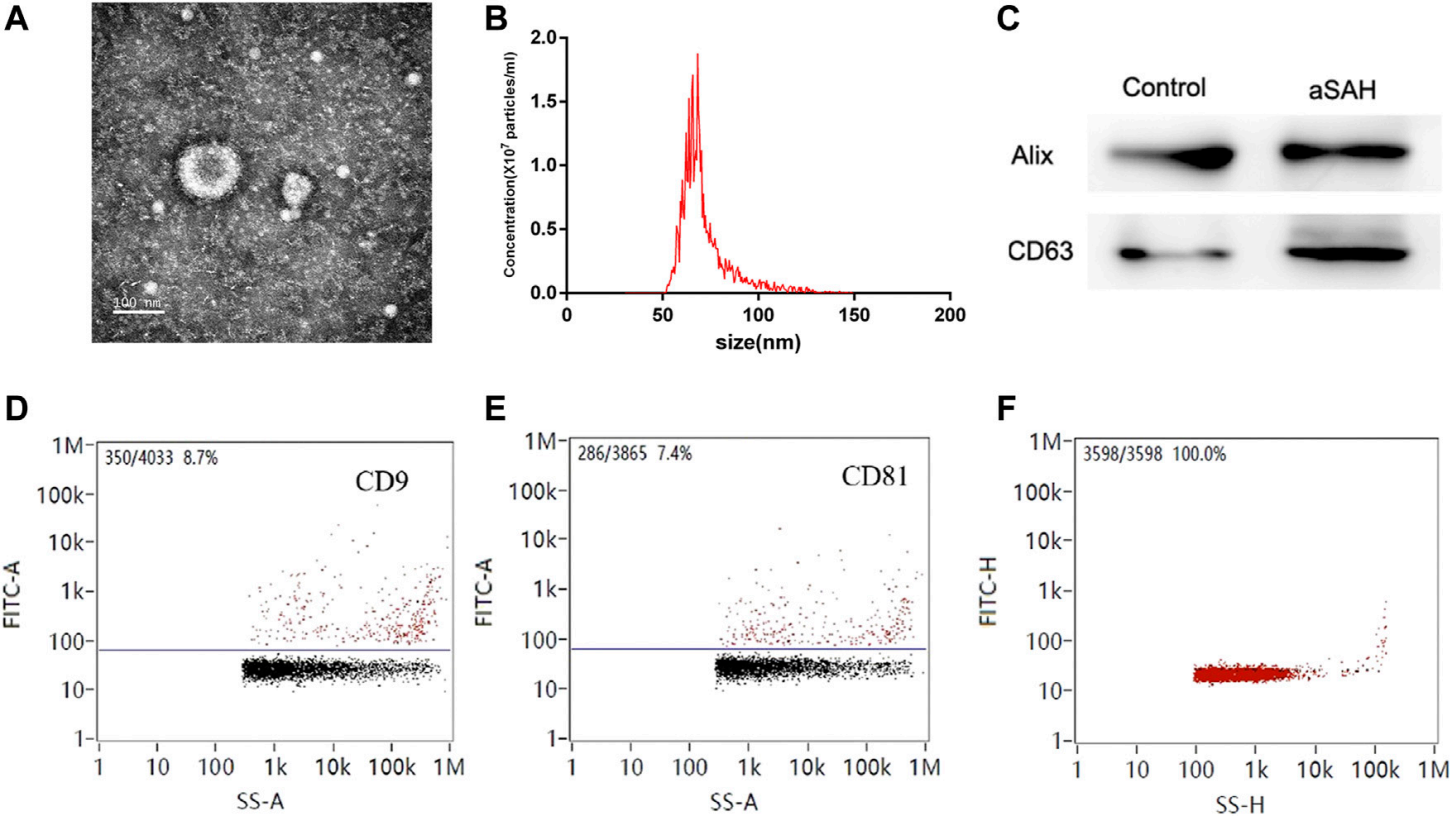
Characterization of exosomes. **(A,B)** Transmission electron microscopy of isolated plasma exosomes. Bar, 100 nm. **(C)** Western blot analysis of Alix and CD63. **(D,E)** NanoFCM analysis of the positive rate of CD9 and CD81. **(F)** NanoFCM analysis of exosome concentration.

TEM was used to evaluate exosome morphology. Exosomes had spherical shapes with sizes of 68.25 ± 13.70 nm and were surrounded by membranes (Figures 2A, B). The levels of CD63 and Alix were more highly expressed in aSAH than in healthy controls (Figure 2C). The identity of exosomes was further validated by quantitating the exosome membrane-associated markers CD9 and CD81 using NanoFCM, and the positivity rates were 8.7% and 7.4% (Figures 2D, E). The sample concentration of exosomes was 9.18 × 10^7^ particles/mL (Figure 2F). There were no obvious differences in size or shape of exosomes between the control and aSAH samples. The quantity and purity of total RNA isolated from exosomes were analyzed on a Bioanalyzer 2100 instrument (Agilent, CA, USA) with RIN number <7.0.

### Distinct plasma exosomal miRNA profiles in aSAH patients

**FIGURE 3:**
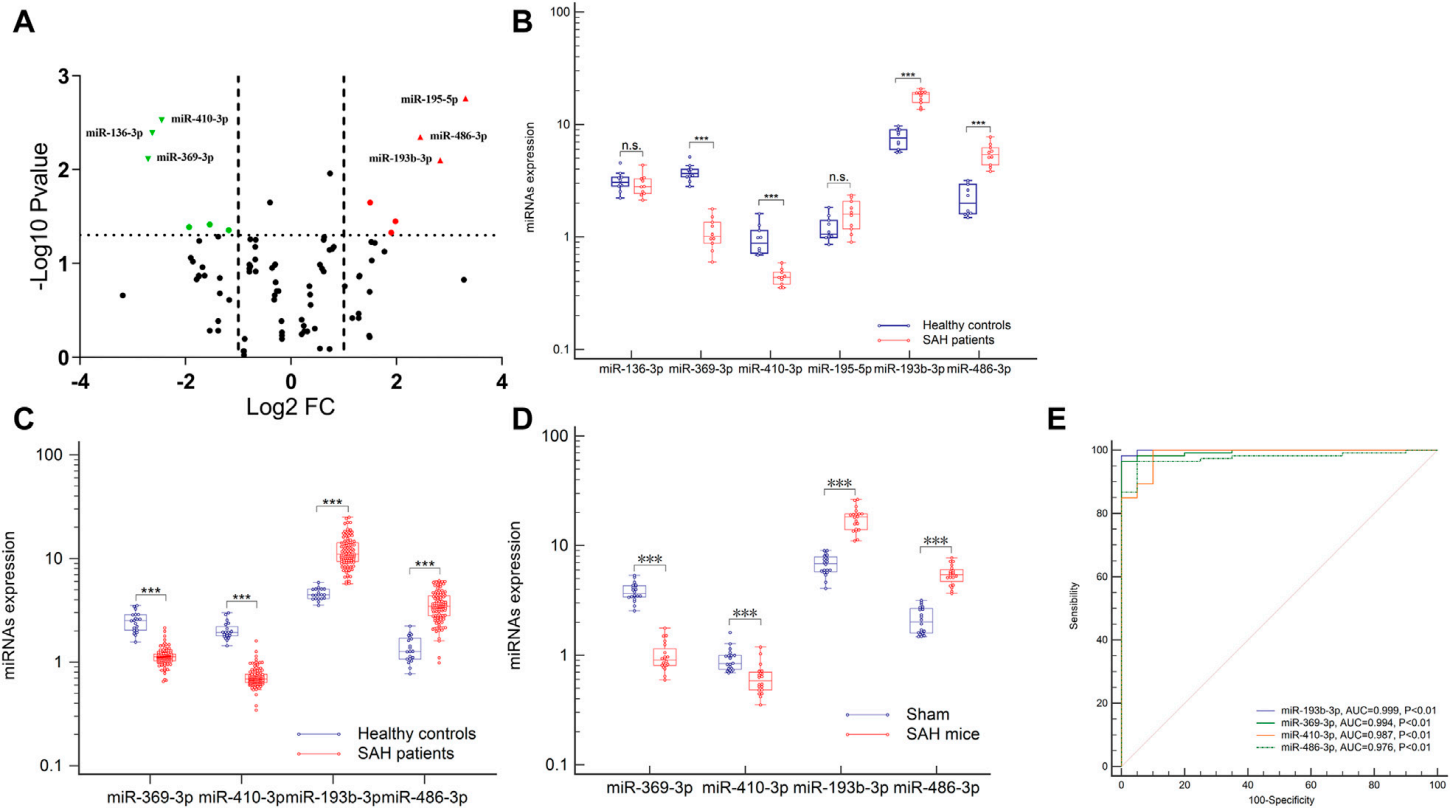
Expression profiles of the circulating exosomal miRNAs after aSAH. **(A,B)** Six differentially miRNAs of NGS were validated in aSAH patients and healthy controls. **(C,D)** Expression of four miRNAs in aSAH (patients, C), (mice, D), and healthy controls. **(E)** ROC curves to distinguish aSAH patients from healthy controls. ***p < 0.001.

To identify potential biomarkers, six plasma samples were analyzed, including 10 control individuals and 10 patients with aSAH. NGS of these samples yielded 9–15 million reads, corresponding to more than 50,000 different RNA sequences. These were aligned to the reference human genome sequence. Only 746 miRNAs from the 2,139 known miRNAs were considered to be expressed, with raw read count ≥1 in at least one sample. Distinct profile expression was found after measuring the expression spectra of six plasma samples (Supplementary Data; Additional Supplementary File S1). To analyze the differences between the groups, a global statistical analysis was used to detect different miRNA sequences (adjusted *p*-value <0.05, |log2 (fold change) | > 2). Six miRNAs were significantly differentially expressed in plasma exosomes of aSAH patients, compared to control samples: hsa-miR-369-3p, hsa-miR-136-3p, hsa-miR-410-3p, hsa-miR-195-5p, hsa-miR-486-3p, and hsa-miR-193b-3p (Figure 3A and Supplementary Table S1). These data suggest that NGS helped us to identify a group of differentially expressed plasma exosomal miRNAs in aSAH patients.

### Validation of NGS data by RT-qPCR in an independent patient cohort

To analyze the miRNA NGS results, 20 total serum samples (10 aSAH and 10 healthy controls) were selected. Real-time qPCR and genomic analysis determined that six circulating exosomal miRNAs were differentially expressed and that four of those miRNAs (hsa-miR-369-3p, hsa-miR-410-3p, hsa-miR-193b-3p, and hsa-miR-486-3p) showed significant differential expression in two groups (Figures 3A, B).

Subsequent technical confirmation of the significance of the observed differences in miRNA expression, with additional RT-qPCR assays on a larger group of samples (113 aSAH and 40 healthy controls), confirmed that hsa-miR-369-3p, hsa-miR-410-3p, hsa-miR-193b-3p, and hsa-miR-486-3p exhibited significantly altered expression levels after aSAH as compared with healthy controls (Figure 3C). To further explore the significance of the observed differences in exosomal miRNA expression, we performed ROC analysis and found that all these miRNAs provided the best AUCs for discriminating between aSAH patients and controls (Figure 3E).

### miRNA biomarker correlation with clinical activity and clinical outcomes

**FIGURE 4:**
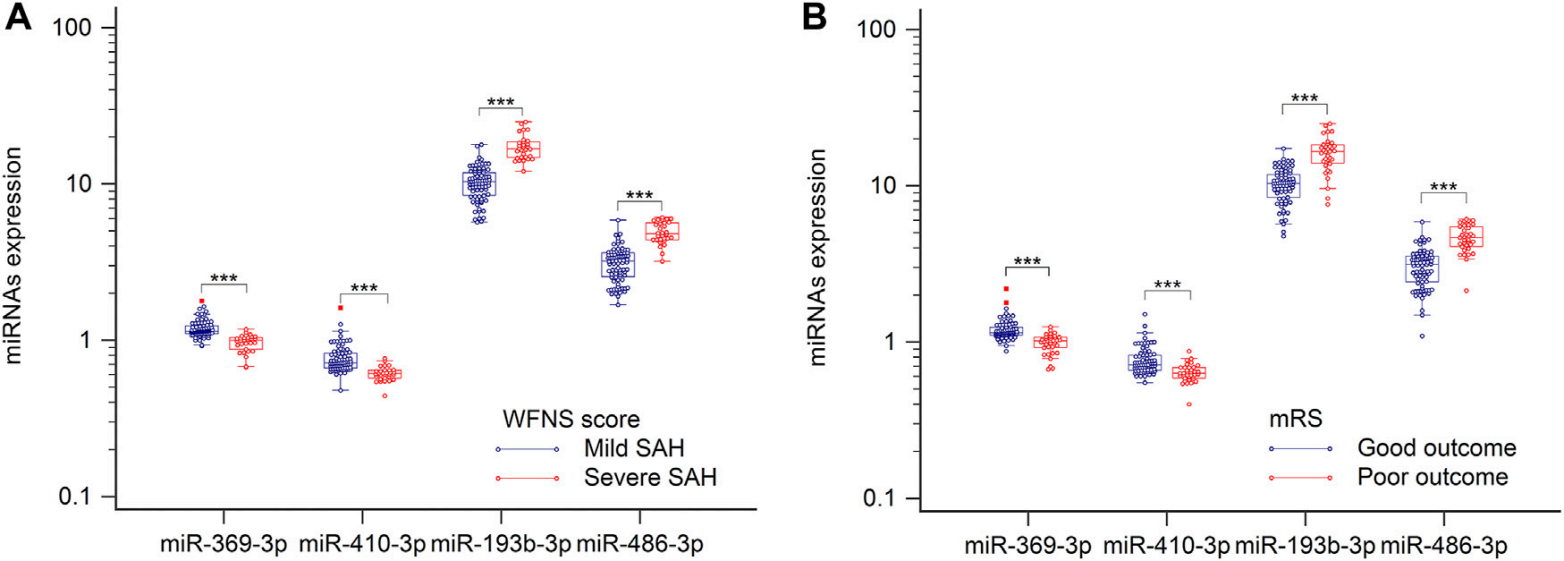
Plasma samples were collected, and miRNAs, analyzed by RT-qPCR. **(A)** Relative levels of miR-369-3p, miR-410-3p, miR-193b-3p, and miR-486-3p in patients with severe SAH and those with mild SAH. **(B)** Relative levels of four miRNAs in relation to clinical outcomes. ***p < 0.001.

World Federation of Neurosurgical Societies (WFNS) scores were used to assess levels of brain injury after aSAH. Because understanding severity and progression is important for designing future treatments for aSAH patients, these miRNAs levels were analyzed with respect to various groups classified according to disease severity. When admitted to the hospital, patients with WFNS grades I–III were classified as mild, and those with grades IV–V were classified as severe (Luo et al., 2022). As illustrated in Figure 4A, comparison of the severe and mild aSAH patients revealed that the miRNA expression levels of hsa-miR-193b-3p and hsa-miR-486-3p were significantly elevated and that levels of hsa-miR-369-3p and hsa-miR-410-3p were significantly lowered (*p* < 0.001). It is important to determine the potential outcomes of aSAH patients at the earliest stages to optimize their treatment. Therefore, the patients were divided into two groups according to their clinical outcomes. As shown in Figure 4B, we clearly see that the levels of hsa-miR-193b-3p and hsa-miR-486-3p were lower in the positive-outcome group than those in the poor-outcome group. However, the levels of hsa-miR-369-3p and hsa-miR-410-3p showed opposite results (both *p* < 0.001). These results may improve the determination of the expression of these miRNAs.

**FIGURE 5:**
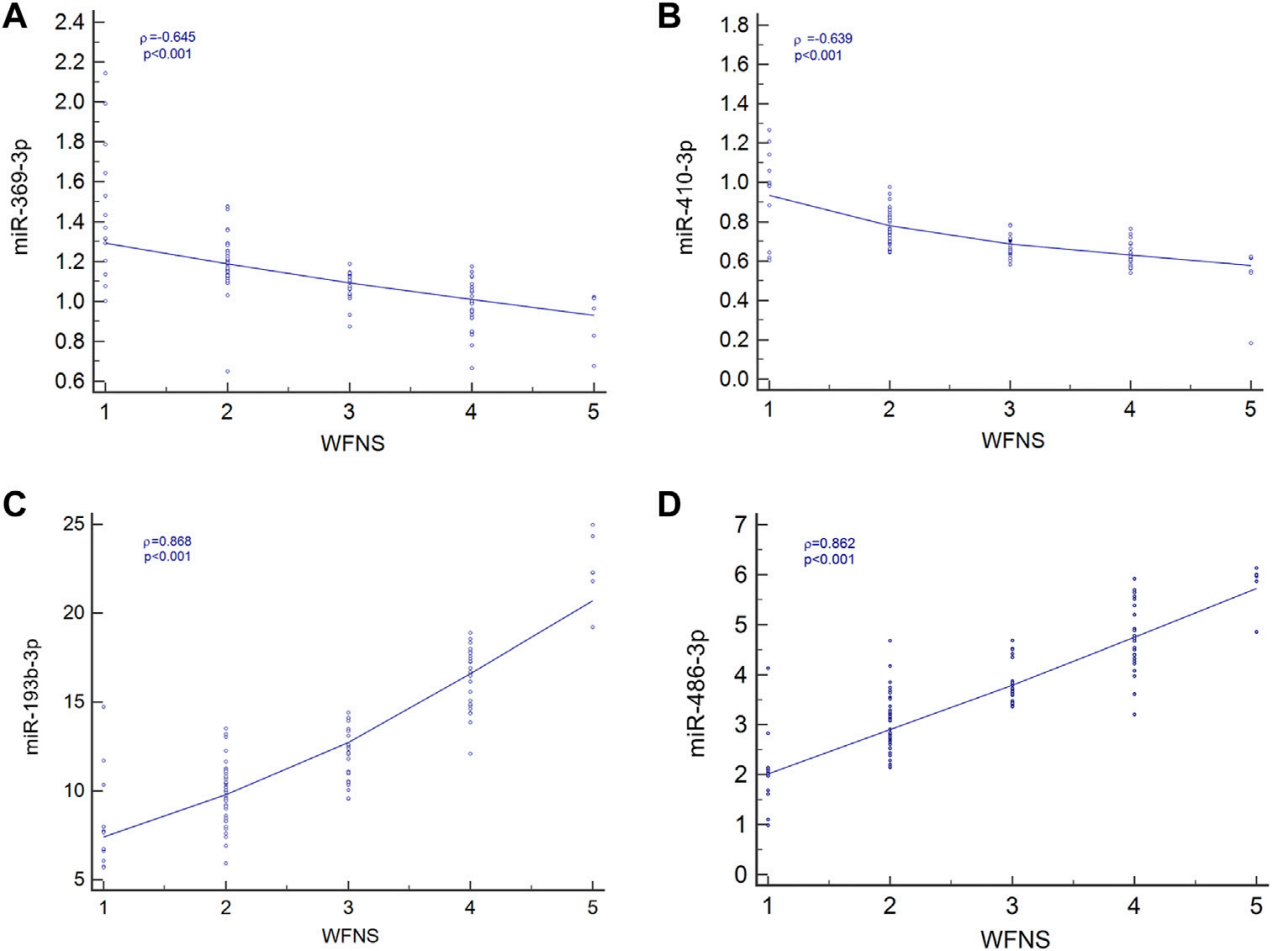
Relationship between four miRNA levels and WFNS grade. **(A)** Relationship between miR-369-3p, **(A)**; miR-410-3p, **(B)**; miR-193b-3p, **(C);** and miR-486-3p, **(D)** levels and WFNS grade.

Spearman’s correlation coefficient analysis was used to investigate the relationships between the four miRNA levels and WFNS grades. The results revealed that in the aSAH patients, plasma exosomal levels of hsa-miR-369-3p (*ρ* = −0.645; *p* < 0.001; 95% CI: −0.753 to −0.541), hsa-miR-410-3p (*ρ* = −0.639; *p* < 0.001; 95% CI: −0.727 to −0.499), hsa-miR-193b-3p (*ρ* = 0.868; *p* < 0.001; 95% CI: 0.688–0.839), and hsa-miR-486-3p (*ρ* = 0.862; *p* < 0.001; 95% CI: 0.746–0.871) were closely correlated with aSAH severity, as scored by WFNS grade and is shown in Figure 5.

In univariate analysis, the WFNS grade, Hunt–Hess grade, and Fisher score were identified as prognostic predictive factors 1 year post-aSAH (Table 1). In the multivariate logistic regression models, levels of miR-369-3p (*p* = 0.009), miR-193b-3p (*p* = 0.040), and miR-486-3p (*p* = 0.012) were significantly associated with mRS at 1 year after aSAH. This finding suggests that severe neurological status upon admission and levels of miR-369-3p, miR-193b-3p, and miR-486-3p indicate a high risk of a poor outcome (Table 2).

**TABLE 1:** Clinicopathological features in aSAH patients.

Parameter	mRS 0–2, *n* = 78	mRS 3–6, *n* = 35	P
Gender			0.86
Male	32	15	
Female	46	20	
Age <61	45	16	0.24
Age ≥61	33	19	
Hypertension			0.25
Yes	38	13	
No	40	22	
Smoking			0.50
Yes	25	9	
No	53	26	
WFNS grade I	13	1	<0.01
WFNS grade II	39	3	
WFNS grade III	23	4	
WFNS grade IV	3	21	
WFNS grade V	0	6	
Hunt & Hess grade I	12	0	<0.01
Hunt & Hess grade II	42	5	
Hunt & Hess grade III	23	16	
Hunt & Hess grade IV	1	11	
Hunt & Hess grade V	0	3	
Fisher score I	13	2	<0.01
Fisher score II	27	3	
Fisher score III	31	8	
Fisher score IV	7	22	

The median age of participants was 61 years.

**TABLE 2:** Multivariable logistic analysis of risk factors in patients with SAH at 1 year.

	Parameter	OR (95%CI)	*P*
**Model 1**	WFNS grade	3.93 (1.11–13.92)	0.034
	Hunt & Hess grade	1.35 (0.30–6.07)	0.700
	Modified Fisher score	1.44 (0.51–4.03)	0.488
	miR-369-3p	0.00 (0.00–0.09)	0.009
**Model 2**	WFNS grade	5.05 (1.55–16.51)	0.007
	Hunt & Hess grade	1.52 (0.36–6.37)	0.568
	Modified Fisher score	1.31 (0.50–3.45)	0.586
	miR-410-3p	0.01 (0.00–53.25)	0.289
**Model 3**	WFNS grade	2.98 (0.88–10.08)	0.079
	Hunt & Hess grade	0.88 (0.17–4.54)	0.877
	Modified Fisher score	1.19 (0.46–3.06)	0.726
	miR-193b-3p	1.49 (1.02–2.17)	0.040
**Model 4**	WFNS grade	3.35 (1.04–10.85)	0.043
	Hunt & Hess grade	0.81 (0.16–4.01)	0.800
	Modified Fisher score	1.08 (0.41–2.85)	0.877
	miR-486-3p	5.39 (1.45–20.05)	0.012

### miRNA expression in mice after SAH

To examine the conservation of miRNA expression, it is necessary to determine whether the miRNAs exhibit significantly altered expression levels in mice after SAH, as compared with human levels, to provide a strong theoretical basis for miRNA-based mechanisms of SAH in mice. Plasma was obtained from the model and sham mice, and levels of the four miRNAs were measured. Increased expression of plasma exosomal miR-193b-3p and miR-486-3p remained statistically significant relative to the controls in this analysis, whereas expression levels of miR-369-3p and miR-410-3p were lower than those of the controls (*p* < 0.001). These results were similar to those of human plasma (Figure 3D).

### Identification of the target genes for circulating exosomal miRNAs

**FIGURE 6:**
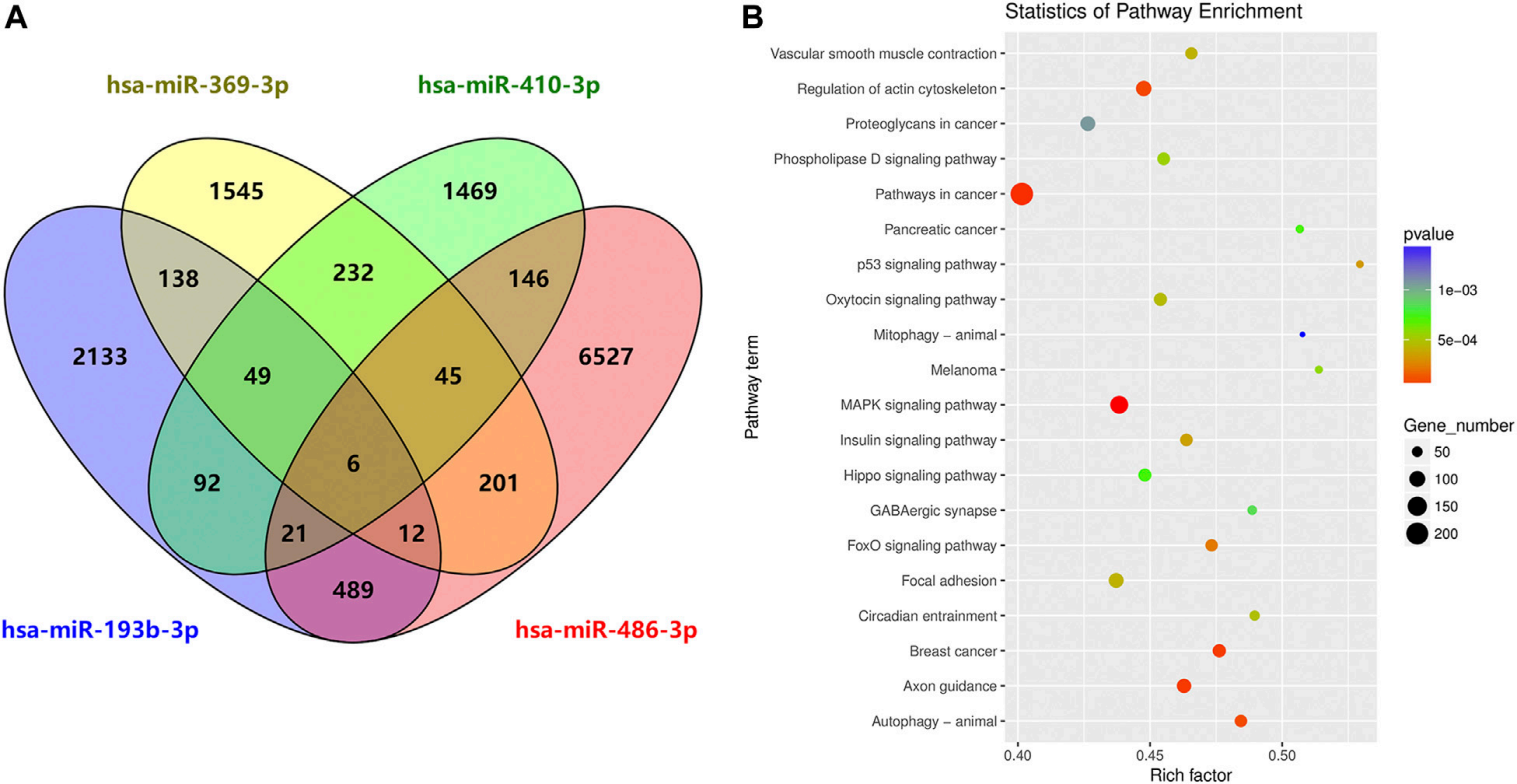
Circulating exosome candidate miRNA target analysis. **(A)** Venn diagram of the overlap of putative targets of miR-369-3p, miR-410-3p, miR-193b-3p, and miR-486-3p. **(B)** KEGG enrichment analysis of putative diseases for these four miRNAs.

To characterize the potential functions of the circulating exosomal miRNAs dysregulated in SAH, we analyzed the potential target genes of miR-369-3p, miR-410-3p, miR-193b-3p, and miR-486-3p in protein-coding transcripts. Considering only strong-evidence targets identified using miRanda and TargetScan Release 7.2, six genes were found to be targeted by all four of these differentially expressed miRNAs (Figure 6A). A list of these factors is provided in Supplementary Table S2.

DIANA Tools provided associations with the KEGG pathway database for each target gene of the four candidate miRNAs. Some identified pathways included terms associated with cancer, the MAPK signaling pathway, focal adhesion, regulation of actin cytoskeleton, axon guidance, autophagy, breast cancer, oxytocin signaling pathway, Hippo signaling, and phospholipase D signaling, among others (Figure 6B).

## Discussion

To the best of our knowledge, this study is not the first to identify exosomal miRNAs as potential biomarkers correlating with disease severity and prognosis in patients with aSAH (Kalani et al., 2020). Recently published work from our laboratory and others has described miRNAs using peripheral blood or cerebrospinal fluid samples of aSAH patients (Powers et al., 2016; Bache et al., 2017; Lai et al., 2017; Lopes et al., 2018); nevertheless, no distinctive signatures have been reported for circulating exosomal miRNAs to date. In this study, we observed that circulating exosomal miRNA expression profiles showed distinct patterns between aSAH patients and HCs. We made three major findings. First, the overall strategy is feasible because exosomal miRNAs were detectable using RT-qPCR on cDNA synthesized from a small volume of patient plasma. Second, levels of circulating exosomal miRNA expression were associated with prognosis in aSAH patients. Finally, an SAH model of C57BL/6 J mice can be used to test clinical assumptions in patients with aSAH.

We began with a total exosomal miRNA analysis, using NGS to identify changes in the expression of miRNAs. We identified six miRNAs that were differentially expressed among the different groups (*p* < 0.05, |log2 (fold change) | > 1 and the expression level was not low). The NGS results for hsa-miR-486-3p, hsa-miR-193b-3p, hsa-miR-369-3p, and hsa-miR-410-3p were verified by RT-qPCR experiments. In the validation set, our results revealed that the levels of the four miRNAs were markedly changed in the aSAH patients, compared to the healthy controls, in association with severity and clinical outcomes. Furthermore, these miRNAs distinguished aSAH patients from healthy controls.

Exosomes provide novel molecular mechanisms of intercellular communication. Importantly, exosome content is not random but rather depends upon the secreted cell’s status (Raposo and Stoorvogel, 2013). In the present study, high levels of miR-193b-3p and miR-486-3p expression occurred more frequently in circulating exosomes from the SAH models. Nevertheless, exosome levels in other tissues were unclear. A previous study showed that the majority of exosomes in circulation originate from other organs because of the tight regulation of the BBB in molecular transport (Kanninen et al., 2016). The results suggest that in patients with SAH, other organs in the body could also secrete exosomes by neurohumoral regulation.

miR-193b-3p has been primarily linked with cancer, chondrocyte metabolism, apoptosis, and autophagy (Meng et al., 2018; Feng et al., 2021; Dinami et al., 2022). Originally, miR-193b-3p was reported to be a tumor suppressor inhibiting several tumor-associated proteins, including the *MYB* oncogene in T-cell acute lymphoblastic leukemia (Mets et al., 2015) and *MORC4* in breast cancer (Yang et al., 2018). In addition, as a novel non-invasive biomarker, extracellular vesicle expression of miR-193b-3p is upregulated in HeLa cancer cells and directly targets HDAC3 (Lin et al., 2018; Meng et al., 2018); it also attenuates neuroinflammation in early brain injury after aSAH in mice (Lai et al., 2020).

miR-486-3p was correlated with central nervous system maturation, inflammation, and cancer (Li et al., 2021; Li et al., 2022; Yu et al., 2022). miR-486-3p has been identified as a candidate diagnostic marker for oral tongue squamous cell carcinoma; in addition, it plays a vital role in neuronal differentiation and central nervous system maturation in the brain (Chen et al., 2017; Yu et al., 2018).

miR-369-3p and miR-410-3p belong to the miR-379-410 cluster, a large genomic miRNA cluster with brain-specific functions located on chromosome 14 in humans and chromosome 12 in mice (Winter, 2015). miR-379-410 cluster miRNAs regulate neurogenesis and neuronal migration in the developing neocortex by targeting N-cadherin, and their levels correlate with glioblastoma aggressiveness and patient survival (Rago et al., 2014; Shahar et al., 2016). miR-369-3p is downregulated in a variety of other solid tumor tissue types and potentially influences cellular function through diverse pathways (Hao et al., 2017; Zou et al., 2018), and miR-410-3p promotes ground-state pluripotency *via* inhibition of multi-lineage differentiation and stimulation of self-renewal in embryonic stem cells (Moradi et al., 2017).

Associations between miRNA and its target genes were explored using miRanda and TargetScan Release 7.2. Considering only strong-evidence targets, six genes were found to be targeted by four miRNAs. RORA is a member of the NR1 subfamily of nuclear hormone receptors involved in circadian rhythm (Zheng et al., 2018). CASK is a calcium/calmodulin-dependent serine protein kinase involved in intellectual disability (Muthusamy et al., 2017). LCOR is a transcriptional corepressor that interacts with estrogen receptor *α* and other nuclear receptors (Cao et al., 2017). ZBED6 is a transcriptional repressor that binds to insulin-like growth factor 2, modulating cell proliferation, wound healing, and neuronal differentiation (Wang et al., 2018), and CNKSR3 is a molecular scaffold that coordinates the assembly of a multiprotein ENaC-regulatory complex and hence plays a central role in sodium homeostasis (Soundararajan et al., 2012).

Conventional neuroimaging (CT, DSA) has obvious advantages in the diagnosis of aSAH; however, due to the complex pathological mechanisms involved in SAH, a considerable number of patients still die from related complications, even after microsurgical treatment. EBI is the key factor influencing the condition changes and prognoses of patients. Unfortunately, the current clinical symptoms combined with neuroimaging cannot accurately predict and evaluate EBI. Therefore, it is urgent to determine EBI treatment significance and positive outcomes of biomarkers. In our previous studies (Lai et al., 2017; Sheng et al., 2018a; Sheng et al., 2018b), we found that serum microRNAs, as non-invasive biomarkers for the presence and progression of subarachnoid hemorrhage, and high levels of miR-502-5p and miR-1297 can predict and evaluate the prognosis of SAH. In addition, Pedrosa et al. (2022) found that the microRNA cerebrospinal fluid profile during the early brain injury period is a biomarker in subarachnoid hemorrhage patients. These findings suggest that circulating miRNAs can be used to assess condition changes and predict prognosis in patients with SAH. In addition, Song et al. 2022) found that miR-340-5p can attenuate EBI caused by SAH-induced neuroinflammation by inhibiting STING. Huang et al. (2023) alleviated EBI after SAH by down-regulating miR-26b expression. These studies provide evidence supporting the treatment of post-SAH complications by miRNA. These results indicate that circulating miRNAs, as non-invasive biomarkers, can be used not only to evaluate condition changes and predict the prognosis of SAH patients but also to alleviate brain damage after SAH, which is beyond the reach of conventional neuroimaging.

In this study, we detected changes in the expression levels of four circulating exosomal miRNAs after SAH; these expression levels can be used to evaluate condition changes and predict prognosis of patients with SAH. Based on previous studies, we can detect the expression level changes of circulating exosomal miRNAs and regulate their levels or tissue distributions to achieve treatment of SAH. In our previous study (Lai et al., 2020), we used targeted delivery of modified Exo/miR-193-3p in brain tissue to alleviate neurobehavioral impairments and neuroinflammation following SAH. Wang et al. (2022) found that exosome-encapsulated microR-140-5p could alleviate neuronal injury by regulating the IGFBP5-mediated PI3K/AKT signaling pathway in SAH. These studies provide broad prospects for the future treatment of SAH by regulating the tissue distribution or modification of circulating exosomal miRNAs for alleviating brain injury after SAH.

There are some potential limitations to our study. First, this was a single-center, retrospective study. Therefore, the results may not be generalizable to populations. Second, the patients underwent surgical treatment or drug therapy prior to serum sample collection, which may have induced changes in expression levels of plasma exosomal miRNAs. Third, clinical parameters such as the Hunt and Hess grades vary between institutions and/or individual clinicians; therefore, the results with this small cohort may reflect biases inherent in the acquisition of such clinical data. Clearly, these results require validation in prospective studies performed on larger cohorts from multicenter clinical trials.

## Conclusion

Circulating exosomal miR-369-3p, miR-410-3p, miR-193b-3p, and miR-486-3p have potential clinical utility as prognostic biomarkers for SAH patients. These findings suggest that peripherally injecting modified exosomes to deliver miRNAs to the central nervous system in future research could be a promising therapy for regulating neuroinflammation or apoptosis.

## Data Availability

The datasets presented in this study can be found in online repositories. The names of the repository/repositories and accession number(s) can be found in: NCBI Gene Expression Omnibus (GEO) (https://www.ncbi.nlm.nih.gov/geo/), GSE222980.
