# Supplementary material for: Diagnosis potential of subarachnoid hemorrhage using miRNA signatures isolated from plasma-derived extracellular vesicles
Source: Front Pharmacol. 2023 Feb 13;14:1090389. doi: 10.3389/fphar.2023.1090389 (PMC9968748; doi:10.3389/fphar.2023.1090389)
Supplement: Supplementary file 1 [file DataSheet1.zip › Supplementary material.docx]

**Supplementary material**

**Diagnosis of subarachnoid haemorrhage using miRNA signatures in nanoparticle isolated plasma extracellular vesicles*miRNA NGS analysis***

*Small RNA library construction and sequencing*

Total exosomal RNA was extracted using Trizol reagent (Invitrogen, CA, USA) following the manufacturer’s procedure. The total RNA quantity and purity were analysis of Bioanalyzer 2100 (Agilent, CA, USA) with RIN number >7.0. Approximately 1 ug of total RNA per sample was used to prepare small RNA library using TruSeq Small RNA Sample Prep Kits (Illumina, San Diego, USA). And then we performed the single-end sequencing (36bp or 50bp) on an Illumina Hiseq2500 at the LC-BIO (Hangzhou, China) following the vendor’s recommended protocol.

*Data Processing*

The raw reads were subjected to an in-house program, ACGT101-miR (LC Sciences,Houston, Texas, USA) to remove adapter dimers, junk, low complexity, common RNA families (rRNA, tRNA, snRNA, snoRNA) and repeats. Subsequently, unique sequences with length in 18~ 26 nucleotide were mapped to specific species precursors in miRBase 21.0 by BLAST search to identify known miRNAs and novel 3p- and 5p- derived miRNAs. Length variation at both 3’ and 5’ ends and one mismatch inside of the sequence were allowed in the alignment.The unique sequences mapping to specific species mature miRNAs in hairpin arms were identified as known miRNAs. The unique sequences mapping to the other arm of known specific species precursor hairpin opposite to the annotated mature miRNA-containing arm were considered to be novel 5p- or 3p derived miRNA candidates. The remaining sequences were mapped to other selected species precursors (with the exclusion of specific species) in miRBase 21.0 by BLAST search, and the mapped pre-miRNAs were further BLASTed against the specific species genomes to determine their genomic locations. The above two we defined as known miRNAs. The unmapped sequences were BLASTed against the specific genomes, and the hairpin RNA structures containing sequences were predicated from the flank 80 nt sequences using RNAfold software (http://rna.tbi.univie.ac. at/cgi-bin/RNAfold.cgi). The criteria for secondary structure prediction were: (1) number of nucleotides in one bulge in stem (<=12) (2) number of base pairs in the stem region of the predicted hairpin (>=16) (3) cutoff of free energy (kCal/mol <=-15) (4) length of hairpin (up and down stems + terminal loop >=50) (5) length of hairpin loop (<=20). (6) number of nucleotides in one bulge in mature region (<=8) (7) number of biased errors in one bulge in mature region(<=4) (8) number of biased bulges in mature region (<=2) (9) number of errors in mature region (<=7) (10) number of base pairs in the mature region of the predicted hairpin (>=12) (11) percent of mature in stem (>=80).

*Analysis of Diﬀerential expressed miRNAs*

miRNA differential expression based on normalized deep-sequencing counts was analyzed by selectively using Fisher exact test, Chi-squared 2X2 test, Chi-squared nXn test, Student t test, or ANOVA based on the experiments design. The significance threshold was set to be 0.01 and 0.05 in each test.

*The Prediction of Target Genes of miRNAs*

To predict the genes targeted by differentially expressed miRNAs, two computational target prediction algorithms (TargetScan 7.0 and miRanda 3.3a ) were used to identify miRNA binding sites. Finally, the data predicted by both algorithms were combined and the overlaps were calculated. The GO terms and KEGG Pathway of these differentially expressed miRNA targets were also annotated.

Find a common set of sequences among all samples. Construct a reference data set. Each data in the reference set is the copy number median value of a corresponding common sequence of all samples. Perform 2-based logarithm transformation on copy numbers (log2(copy#)) of all samples and reference data set. Calculate the (log2(copy#) difference (∆(log2(copy#)) between individual sample and the reference data set. Form a subset of sequences by selecting|∆(log2(copy#)| <2, which means less than (2^2^ =)4 fold change from the reference set. Perform linear regressions between individual samples and the reference set on the subset sequences to derive linear equations y = a_i_x + b_i_， where a_i_ and b_i_ are the slop and interception, respectively, of the derived line, x is log2(copy#) of the reference set, and y is the expected log2(copy#)of sample i on a corresponding sequence. Calculate the mid value xmin = max(x)-min(x) /2of the reference set. Calculate the corresponding expected log2(copy#) of sample i, y_i,mid_ = a_i_x_mid_ + b_i_. Let y_r,mid_ = x_mid_, let ∆ y_i_ = y_r,mid_-y_i,mid_, which is the logarithmic correction factor of sample i. We then derive the arithmetic correction factor f_i_ = 2^Δyi^ sample i. Correct copy numbers of individual samples by multiplying corresponding arithmetic correction factor fi to original copy numbers.

*RNA Isolation*

Total RNA was extracted from exosome using the exoRNeasy Serum/Plasma Midi Kit (for exosome, Qiagen, Valencia, CA) and QIAzol (for tissues, Qiagen, Valencia, CA) according to the manufacturer’s instructions. Briefly, QiAzol (700 µL, Qiagen) was added to a volume of exosome and vortex for 5 min. An external control (cel-miR-39 [Qiagen, Valencia, CA]) for miRNAs was mixed with samples prior to miRNA extraction. The aqueous phase containing total RNA was extracted with chloroform (90µl) and transferred to an RNeasy minElute Spin Column. The bound RNA was washed with Buffer RWT and Buffer RPE. miRNAs were finally eluted in RNase-free water (14 µL), quantified with the NanoDrop 2000 spectrophotometer (NanoDrop Technologies, Houston, Texas, USA), and stored at -80°C. Only samples with an OD A260/A280 ratio between 1.8 and 2.0 were utilized for further analysis by RT-PCR.

*Reverse transcription (RT)*

cDNA was synthesized using the miRcute Plus miRNA First-Strand cDNA Synthesis Kit (Tiangen Biotech). Each reaction mixture utilized 2 × miRNA RT Reaction Buffer (10 μl), 2 μl miRNA RT Enzyme Mix, 4 μl total RNA and 4 μl RNase-Free ddH2O to a reaction volume to 20 μl. Then, with the following cycle profile: 42˚C for 60 min, and 95˚C for 3 min. Synthesized cDNA was stored at –20˚C for further analysis.

*Reverse transcription quantitative real-time PCR (RT-qPCR)*

Real-time PCR was performed using the miRcute Plus miRNA qPCR Detection kit (SYBR Green) on CFX96 TouthTM Real-Time PCR Detection Systems (Bio-rad Lab, Inc., Hercules, California, U.S.A.), using 2 μl forward primer(Catalog no.MS00006853 for miR-369-3p, MS00009765 for miR-410-3p, MS00031605 for miR-195-5p, MS00007714 for miR-193b-3p, MS00004144 for miR-486-3p, MS00008645 for miR-136-3p), specific to the external control (cel-miR-39), 10 μl 2 × miRcute Plus miRNA Premix (with SYBR and ROX), 0.4 μl reverse primer (10 μM), 2 μl cDNA and 5.6 μl ddH2O to a final volume of 20 μl (all from Tiangen Biotech). A total volume of 20 μl per reaction was transferred to 96-well plates and incubated for 15 min at 95°C, followed by 5 cycles at 94°C for 25 sec, 65°C for 30 sec and 72°C for 34 sec, then 45 cycles at 94°C for 20 sec, 60°C for 34 sec. All samples were run in triplicate.

Samples with cycle threshold (Ct) over 30 were regarded as having no expression. The relative miRNA level was expressed as2 ^-△Ct^, where the raw data of the target miRNA were normalized to the Ct of the external control. PCR products were separated by agarose gels to determine the product size, and dissociation curves were used to examine the specificity of the qPCR assay.
