# Supplementary material for: Diagnosis potential of subarachnoid hemorrhage using miRNA signatures isolated from plasma-derived extracellular vesicles
Source: Front Pharmacol. 2023 Feb 13;14:1090389. doi: 10.3389/fphar.2023.1090389 (PMC9968748; doi:10.3389/fphar.2023.1090389)
Supplement: Supplementary file 1 [file DataSheet1.zip › Supplementary tables.docx]

**Supplementary table 1. Selected differentially expressed miRNAs in the screening phase**

| miRNA name | log2(Fold Change) | P value | Expression in SAH(vs control) |
| --- | --- | --- | --- |
| hsa-miR-369-3p hsa-miR-136-3p  hsa-miR-410-3p hsa-miR-195-5p  hsa-miR-486-3p  hsa-miR-193b-3p | -1.17  -1.06  -1.55  1.03  1.03  1.08 | 3.62E-02  4.15E-02  7.91E-03  2.73E-02  1.84E-02  1.81E-02 | Down-regulated  Down-regulated  Down-regulated  Up-regulated  Up -regulated  Up -regulated |

**Supplementary** **table 2. A list of the individual putative overlapping targets of four miRNAs**

| Gene Symbol | Gene Annoation |
| --- | --- |
| RORA CASK  LCOR  GPALPP1  ZBED6  CNKSR3 | RAR related orphan receptor A  Calcium/calmodulin dependent serine protein kinase  Ligand dependent nuclear receptor corepressor  GPALPP motifs containing 1  Zinc finger BED-type containing 6  CNKSR family member 3 |
